# Supplementary material for: Integrated Metabolomics Approach Reveals the Dynamic Variations of Metabolites and Bioactivities in Paeonia ostii ‘Feng Dan’ Leaves during Development
Source: Int J Mol Sci. 2024 Jan 15;25(2):1059. doi: 10.3390/ijms25021059 (PMC10816844; doi:10.3390/ijms25021059)
Supplement: Supplementary file 1 [file ijms-25-01059-s001.zip › supplementary material 1.pdf]

Table S1. Sampling dates and corresponding important phenology of *P. ostii* 'Feng Dan' leaves during development.

Code	Date	Phenology	Code	Date	Phenology
S1	March 7th	bud lifting	S10	June 8th	seed nutrient accumulation
S2	March 17th	wind bell-like flower bud	S11	June 19th	seed nutrient accumulation
S3	March 27th	flat peach-like flower bud	S12	June 29th	seed nutrient accumulation
S4	April 7th	flower blooming	S13	July 8th	seed nutrient accumulation
S5	April 18th	flower failure	S14	July 19th	seed coloring
S6	April 28th	seed expansion	S15	July 29th	seed maturity
S7	May 9th	seed expansion	S16	August 10th	seed abscission
S8	May 18th	seed expansion	S17	August 18th	leaves began to turn yellow
S9	May 28th	seed expansion			

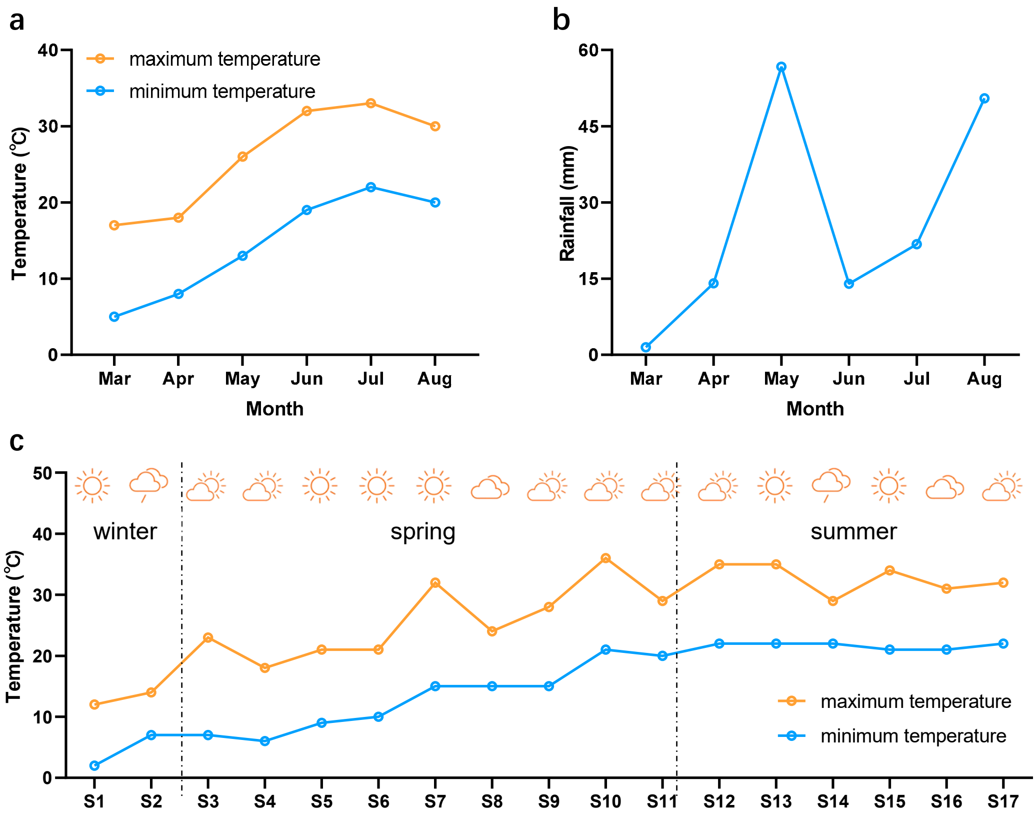

Figure S1. Environmental factors of *P. ostii* 'Feng Dan' leaves during developmental stages.
